# *Notes from the Field:* Contact Tracing Investigation after First Case of Andes Virus in the United States — Delaware, February 2018

**DOI:** 10.15585/mmwr.mm6741a7

**Published:** 2018-10-19

**Authors:** Aaron Kofman, Paula Eggers, Anne Kjemtrup, Rebecca Hall, Shelley M. Brown, Maria Morales-Betoulle, James Graziano, Sara E. Zufan, Shannon L.M. Whitmer, Deborah L. Cannon, Cheng-Feng Chiang, Mary J. Choi, Pierre E. Rollin, Martin S. Cetron, Hayley D. Yaglom, Monique Duwell, David T. Kuhar, Melissa Kretschmer, Barbara Knust, John D. Klena, Francisco Alvarado-Ramy, Trevor Shoemaker, Jonathan S. Towner, Stuart T. Nichol

**Affiliations:** ^1^Epidemic Intelligence Service, CDC; ^2^Division of High-Consequence Pathogens and Pathology, National Center for Emerging and Zoonotic Infectious Diseases, CDC; ^3^Division of Public Health, Delaware Department of Health and Social Services; ^4^California Department of Public Health; ^5^Division of Global Migration and Quarantine, CDC; ^6^Arizona Department of Health Services; ^7^Maryland Department of Health; ^8^Division of Healthcare Quality Promotion, National Center for Emerging and Zoonotic Infectious Diseases, CDC; ^9^Maricopa County Department of Public Health, Phoenix, Arizona.

In January 2018, a woman admitted to a Delaware hospital tested positive for New World hantavirus immunoglobulin M (IgM) and immunoglobulin G (IgG) by enzyme-linked immunosorbent assay (ELISA). Subsequent testing by CDC’s Viral Special Pathogens Branch detected New World hantavirus by nested reverse transcription–polymerase chain reaction (RT-PCR) and Andes virus by nucleic acid sequencing. This case represents the first confirmed importation of Andes virus infection into the United States; two imported cases have also been reported in Switzerland (*1*). Before her illness, the patient had traveled to the Andes region of Argentina and Chile from December 20, 2017, to January 3, 2018. She stayed in cabins and youth hostels in reportedly poor condition. No rodent exposures were reported. After returning to the United States on January 10, she developed fever, malaise, and myalgias on January 14. On January 17, while ill, she traveled on two commercial domestic flights. She was hospitalized during January 20–25 in Delaware and discharged to her home after clinical recovery.

Andes virus, a species of New World hantavirus, is transmitted to humans primarily through contact with long-tailed rice rats (*Oligoryzomys longicaudatus*), which are endemic to much of Argentina and Chile. Clinical symptoms are similar to those of other New World hantaviruses, and the case fatality rate is approximately 36% (*2*). Unlike all other hantavirus species, Andes virus can be transmitted from person to person; however, transmission is typically limited to close contacts of ill persons (*2*–*4*). Because of this risk, a contact tracing investigation was initiated by CDC as well as state and county health departments.

A suspected case was defined as the occurrence of one or more of the following signs or symptoms in a person with close contact with the patient within 42 days (the maximum incubation period) after last contact: new onset anorexia, chest pain, cough, diarrhea, fever, headache, muscle pain, nausea, or vomiting. A high-risk contact was defined as a person with exposure to the traveler’s body fluids. A low-risk contact was defined as a person who, in the absence of exposure to body fluids, provided medical care or in-flight service to, or was seated near, the traveler for at least 1 hour.

Among 53 contacts identified in six states, 51 were successfully contacted (Table). Of these, 28 were health care personnel, 15 were airline contacts (flight crew who served the traveler and passengers seated within one seat of the traveler), and eight were other contacts of the traveler (including acquaintances and a hospital roommate). All contacts were advised to self-monitor their temperature daily for 42 days from last contact and to seek medical evaluation for any of the specified symptoms. Contacts who developed symptoms were tested for hantavirus by RT-PCR and serology by CDC’s Viral Special Pathogens Branch.

**TABLE:** Number and types of contacts traced during Andes virus investigation, by state — United States, 2018

State	Airline contacts	Health care contacts	Other contacts	Total contacts (no. contacted)	High-risk contacts	Specimens sent to CDC for testing
Delaware	0	28	9	37 (35)	1	0
**California**	**7**	**0**	**1**	**8 (8)**	**1**	**5**
**Arizona**	**3**	**0**	**0**	**3 (3)**	**0**	**1**
**Maryland**	**2**	**0**	**0**	**2 (2)**	**0**	**0**
**Pennsylvania**	**2**	**0**	**0**	**2 (2)**	**0**	**0**
**Illinois**	**1**	**0**	**0**	**1 (1)**	**0**	**0**
Total	15	28	10	53 (51)	2	6

Two high-risk contacts were identified: a health care worker with exposure to the traveler’s sweat and a family member with exposure to the traveler’s clothes and bedding. Both high-risk contacts remained asymptomatic. Six low-risk contacts, all flight attendants, reported influenza-like illness, diarrhea, or mild rhinitis during the incubation period; all tested negative for hantavirus by RT-PCR and serology. The remaining low-risk contacts remained asymptomatic, and the investigation concluded on March 8.

Hospitalized patients with Andes virus should be managed with standard contact and droplet precautions. Although the risk for person-to-person Andes virus transmission is low, contact tracing should be considered to identify potential cases and limit additional exposures. Health care personnel should consider Andes virus in returning travelers with nonspecific febrile illness or acute respiratory disease whose travel history includes the Andes region of Argentina or Chile in the preceding 6 weeks.
